# Data-Fusion-Based Quality Enhancement for HR Measurements Collected by Wearable Sensors

**DOI:** 10.3390/s24102970

**Published:** 2024-05-07

**Authors:** Shenghao Xia, Shu-Fen Wung, Chang-Chun Chen, Jude Larbi Kwesi Coompson, Janet Roveda, Jian Liu

**Affiliations:** 1 Statistics GIDP, Department of Mathematics, University of Arizona, Tucson, AZ 85721, USA; shenghaoxia@arizona.edu; 2 Department of System and Industrial Engineering, University of Arizona, Tucson, AZ 85721, USA; 3 College of Nursing, University of Arizona, Tucson, AZ 85721, USA; wung@arizona.edu; 4 Department of Electrical and Computer Engineering, University of Arizona, Tucson, AZ 85721, USA; changchunchen19@arizona.edu (C.-C.C.); jcoompson@arizona.edu (J.L.K.C.); meilingw@arizona.edu (J.R.)

**Keywords:** wearable sensors, precision care, functional data analysis, Bayesian inference, Gaussian process, data quality, heart rate patterns, personalized patterns

## Abstract

The advancements of Internet of Things (IoT) technologies have enabled the implementation of smart and wearable sensors, which can be employed to provide older adults with affordable and accessible continuous biophysiological status monitoring. The quality of such monitoring data, however, is unsatisfactory due to excessive noise induced by various disturbances, such as motion artifacts. Existing methods take advantage of summary statistics, such as mean or median values, for denoising, without taking into account the biophysiological patterns embedded in data. In this research, a functional data analysis modeling method was proposed to enhance the data quality by learning individual subjects’ diurnal heart rate (HR) patterns from historical data, which were further improved by fusing newly collected data. This proposed data-fusion approach was developed based on a Bayesian inference framework. Its effectiveness was demonstrated in an HR analysis from a prospective study involving older adults residing in assisted living or home settings. The results indicate that it is imperative to conduct personalized healthcare by estimating individualized HR patterns. Furthermore, the proposed calibration method provides a more accurate (smaller mean errors) and more precise (smaller error standard deviations) HR estimation than raw HR and conventional methods, such as the mean.

## 1. Introduction

Population aging is a worldwide fundamental concern. Both the number and percentage of older adults are rising in every nation in the world. According to the World Social Report 2023, the number of older adults in the world is projected to increase from 761 million in 2021 to 1.6 billion in 2050 [1]. Older adults are a particularly vulnerable population with the potential for severe physical and economic limitations and drastically deteriorating self-sufficiency. In addition, chronic multiple conditions, such as heart disease, diabetes, and cancer, are common in older adults. These complex conditions require specialized healthcare services to monitor various health statuses, including physical activity level, sleep quality, and fall risk [2], by tracking a variety of biophysiological variables, such as heart rate (HR), blood pressure, and blood glucose. Advanced technologies that aim to maintain older adults’ living independence have emerged as a promising solution to address the healthcare needs of the aging population. These technologies enabled the continuous capture of health-related information in an unobstructed manner, supporting safer independent living as well as the early detection of health changes and care of older adults [3]. Limited to the knowledge, attention, and discipline necessary to follow the management plan that includes monitoring relevant biophysiological variables, many older adults will need the technology of wearable sensors, and the Internet of Things (IoT) to achieve better clinical quality in monitoring their biophysiological variables, leading to an improved quality of life [4].

**Figure 1:**
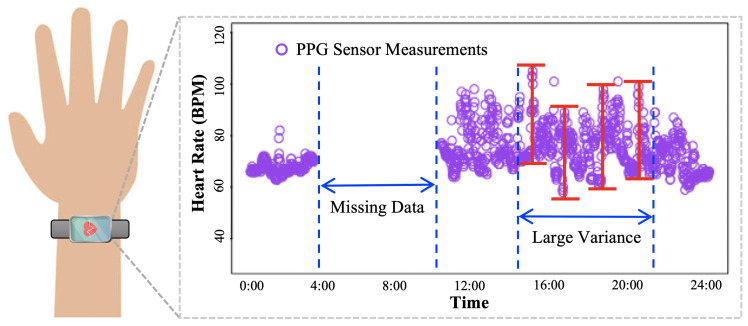
Illustration of a photoplethysmography (PPG) wearable sensor and collected heart rate (HR) data, which include missing data and large variance (red bar illustrates the large variation of heart rate measurements in consecutive timestamps)

Among the biophysiological variables, HR is an essential and widely used indicator of cardiac activity in various physical and health conditions [5], given that HR is strongly positively associated with cardiovascular morbidity and mortality [6]. Thus, HR is a useful biomarker in monitoring cardiovascular disease and chronic degenerative disease [7], for which older adults are susceptible. The negative impact of these diseases is not limited to affecting the emotional and physical integrity of older adults, deriving a series of fatal collateral consequences, such as falls [8]. Individuals who fell are found to have increased variation in their HR and blood pressure than those who did not fall. It suggests that cardiovascular lability may be an influential predictor of falls [9]. Therefore, HR is considered a necessary biophysiological variable to be monitored by wearable sensors equipped with reading technologies, such as photoplethysmography (PPG). Wearable PPG sensors measure HR by photodiodes that capture the changes of light reflection from the microvascular bed of tissue, which quantifies the volumetric variations of blood circulation. As a result, wearable PPG sensors are often designed as wrist devices, as illustrated in Figure 1. Benefiting from this remote continuous HR monitoring by PPG sensors, sequentially measured HR data over time, i.e., HR time series, can be collected for health monitoring in a timely manner.

Although wearable PPG sensors enable the accessibility and remote monitoring of continuous HR, data quality is of major concern due to technological and practical imperfections in real-world applications. The unsatisfactory quality of the data collected from wearable sensors is usually manifested as two unavoidable characteristics of the HR time series: missing data and large variance, as illustrated in Figure 1. Missing data, due to data lost or incomplete data, is prevalent and can be due to various reasons. A few known reasons for missing data include sensor malfunction, lack of sufficient battery power, transmission problems, dropped connections, and problems with sensor synchronization [10]. Published studies rarely report detailed methodology to handle missing data. A commonly used approach tends to focus on the concepts of “valid days” and “numbers of valid days”. For example, researchers may consider the data valid if the device was worn for at least 10 of the 24 h for at least 5 of 7 days [11]. In addition, HR data collected by wearable PPG sensors have large variance, because of their high susceptibility to motion artifacts [12] and external light sources [13]. Specifically, these wearable PPG sensors used LED as a light source and photo detector as a light reflection receiver to measure volumetric changes in blood [14], which can be utilized to calculate the HR. When subjects are in movement, excessive noise will be induced by motion artifacts, typically caused by the displacement of the sensor over the skin, changes in skin deformation, and ambient temperature [14].

The unsatisfactory quality of data poses great challenges to wearable-sensor-based biophysiological monitoring, as the HR analysis relies on complete and precise HR measurements. To overcome these challenges, numerous HR analysis methods have been developed. Traditionally, it is common that specific cut-off values are used to target monitoring, leading to only surrogate values, such as the mean or median, of the intermittently measured HR data being used for interpretation while abandoning the missing HR data [15]. Linear interpolation was commonly used in HR pre-processing to alleviate the impact of missing data by filling the missing data based on a linear regression model estimated from the adjacent HR values [16]. These conventional methods do not consider the temporal pattern of the continuously measured HRs and may yield inaccurate health information.

Continuous HR monitoring enables the understanding and modeling of the temporal patterns embedded in individuals’ HR data, such as daily activities [17], varying emotions [18], and health status fluctuations. Therefore, it is imperative to ensure the quality of HR data and reserve the temporal patterns. Recently, approaches considering the temporal patterns of HR data have become prevalent. The autoregression (AR) model [19] was employed to model the temporal patterns that exist in HR time series, resulting in accurate HR prediction. However, the performance of this type of approach is sensitive to the data quality. Bidirectional long-short-term-memory (Bi-LSTM) neural network and temporal convolutional network (TCN) [20] were adopted to model the HR temporal patterns and estimate the missing data, given a large amount of historical HR for model training. However, these models are only designed to estimate missing values in a short period, such as one cardiac cycle. In contrast, the missing period in continuous HR monitoring is usually much longer in practice. Furthermore, the successful training of the neural network-based models requires a large amount of individuals’ historical HR data that may not be accessible.

Data imputation methods were reported for substituting missing HR data with predicted values. The Gaussian process (GP) [21] was employed to impute the missing HR data and provide personalized monitoring. It has shown that GP is capable of accurately estimating over 24 h of missed HR from wearable devices, and thus, the missing data problem is shown in Figure 1 can be potentially resolved by GP. More importantly, the uncertainties induced by imputation errors can be quantified by GP models to support clinical decision making. For instance, a Gaussian process latent variable model (GPLVM) [22] was proposed to impute the missing HR data. Specifically, a lower-dimensional embedding was learned from a small complete dataset and then used to impute the missing values in the incomplete dataset. Afterward, a support vector machine taking into account the imputation errors was developed to conduct classification tasks, resulting in optimal support vectors and improved classification results. However, GP imputes missing HR data and quantifies uncertainty at each time stamp, leading to a prohibitive computational burden. Furthermore, the GP models the temporal pattern of HR with pre-defined covariance functions, such as linear, exponential, and radial basis functions, which limit the compatibility to model sophisticated correlation embedded in HR time series [23]. Furthermore, GP models are susceptible to excessive noise induced by motion artifacts, leading to inaccurate mean HR modeling and estimation.

**Figure 2:**
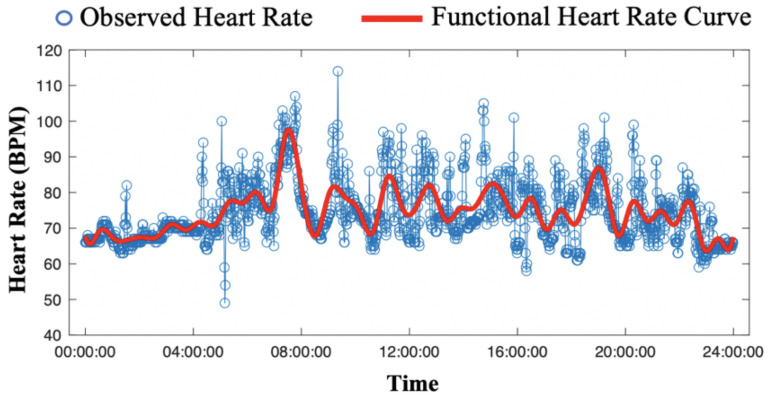
Comparison of the raw heart rate (HR) measurements using photoplethysmography with excessive noise induced by motion artifacts (in blue), and the smoothed HR time series by the functional data analysis (in red).

In real-world scenarios, physical activities are inevitable, and the problem raised by motion artifacts needs to be addressed to enhance the quality of the data measured by wearable sensors. Filtering was commonly used to remove the motion artifacts in HR pre-processing [24]. However, this method treated each HR data point (the HR measured at a particular time stamp) independently and ignored the overall temporal patterns of the continuously measured HR. Functional data analysis (FDA) [25] methods were developed to model the HR time series with a functional HR curve represented by a linear combination of basis functions in lower dimensions, resulting in lower computational costs and dimensional reduction. In addition, FDA approximates the HR time series with a smooth curve, considering the overall temporal patterns. This feature enables the removal of potential outliers induced by motion artifacts, as illustrated in Figure 2. Although FDA is effective in modeling the time series data and reducing noise, its model estimation is significantly affected by the presence of missing HR data.

To enhance the quality of HR data collected from wearable sensors for continuous physiological status monitoring, this paper proposes an FDA-based GP model, named basis-expansion-based GP (BEGP), to learn HR temporal patterns. BEGP combines GP and FDA, using the basis expansion of FDA to model the mean function and covariance function in a GP model. The unique BEGP model enables the imputation of the missed HRs and the reduction in the excessive variance induced by motion artifacts. The missing HR values and their uncertainty are approximated by mean function and covariance function of GP, respectively, which are modeled by FDA. Furthermore, a Bayesian inference framework is developed to calibrate the HR temporal patterns by fusing the historical HR temporal patterns and newly collected HR measurements.

The rest of the article is structured as follows. Section 2 describes the conducted experiment, and methods proposed to remove motion artifacts and calibrate HR temporal patterns. Section 3 presents the experimental results for validating the proposed method, followed by a discussion in Section 4. Finally, conclusions are provided in Section 5.

## 2. Materials and Methods

### 2.1. Participants and Experimental Equipment

In this research, we recruited 10 older adults residing in assisted living or home settings and conducted extensive data collection, and the subjects’ demographics and health conditions are shown in Table 1. Our research team meticulously recorded an array of biophysiological measurements by distinct sensors, including HR sensors, Garmin Vivoactive 4/4S (Garmin Corporation, New Taipei City, Taiwan), Polar H10 (Polar Electro Oy, Kempele, Finland); glucose monitor sensor, FreeStyle Libre 2 (Abbott Laboratories, Chicago, IL, USA); and blood pressure sensor, Omron (Omron Corporation, Kyoto, Japan), aimed for health status monitoring. Among these, we selected the Polar H10 and Garmin watch, which measured HR in this analysis. Data collected by Polar H10 and Garmin were downloaded via ECG logger for Polar H10 by Matti Mononen, version 2.3 (via Google Play), and Labfront (PhysioQ, Boston, MA, USA), respectively. The Garmin Vivoactive 4/4S, worn on the non-dominant hand, delivered HR data every second. Garmin relies on PPG for HR measurement, a method susceptible to motion artifacts, consequently yielding less precise HR measurements. Meanwhile, the Polar H10, a wearable chest strap HR sensor, measured HR using a single lead electrocardiogram (ECG) which offered high precision and accuracy. The recorded ECGs were inspected by cardiovascular clinicians to remove artifacts that may affect HR measurements, via Python version 3.11.2 with Nerokit2 toolbox [26]. Hence, we employed Garmin as the primary data source for HR measurement, with the Polar H10 serving as a supplementary means for data validation. Our collected data indicate that the Garmin collected more comprehensive HR measurements compared to the Polar H10. The detailed missing data rate and days of data collection for 10 subjects (older adults) are listed in Table 2. HR measurements were randomly lost due to issues such as sensor misplacement, battery drain, and unwillingness to wear the sensor. Furthermore, caregivers spent an average of one hour charging the Garmin each day. Once the wearable sensor stopped recording the data, a continuous time interval of data was missed before the issue was fixed. Therefore, the missing data are randomly distributed over time and manifested as losses of continuous time intervals. The low missing data rate from Garmin provided complete information on individual-specific HR patterns, suggesting that the Garmin wearable watch is appropriate for continuous and long-term monitoring of HR data; while measurements collected from Polar H10 served as HR ground truth in evaluating the accuracy of calibrated HR. All the HR data analysis was conducted in R version 4.3.1.

To alleviate the impact of motion artifacts and noise of Garmin HR measurements, denoising, and calibration approaches have been developed to improve the data quality of Garmin HR measurements. Section 2.2 introduces basis expansion for motion artifact removal in Garmin HR measurements. Section 2.3 proposes a BEGP for calibrating HR patterns by fusing the historical HR patterns and newly collected HR measurements.

### 2.2. Motion Artifacts Removal by Basis Expansion

**Figure 3:**
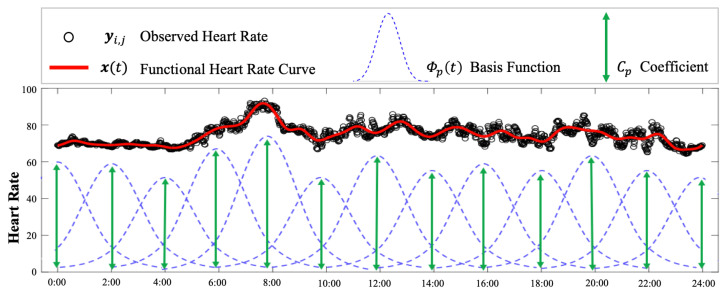
An illustration of the modeling heart rate (HR) by basis expansion. The functional HR curve is an approximation of the HR time series with motion artifacts removed.

In this paper, HR time series were assumed to be continuous functions denoted by x(t), where $t\in [0,T_{period}]$ is the time, $T_{period}$ represents the time period, such as one day. In this paper, $T_{period}=24$ h because we studied the diurnal HR pattern of older adults. The continuous HR data time series x(t) is not directly observable, while a series of data points $\left[t_{i,j},y_{i,j}\right]$ were measured by sensors, where $t_{i,j}$ denotes the *j*th timestamp on the *i*th day and $y_{i,j}$ is the corresponding measurements of $x(t_{i,j})$; i=1,2,3,...,M represents the index of days, and M is the total number of days; $j=1,2,...,N_{i}$ denotes the index of timestamps and $N_{i}$ denotes the total number of timestamps in which the HR variable is measured on *i*th day. However, the HR measurements may be contaminated by errors induced by the imprecision of sensors and motion artifacts, and thus, $y_{i,j}$ can be represented as a summation of the true HR value $x(t_{i,j})$ and measurement error $\varepsilon_{i,j}$, as shown in (1), where $\varepsilon_{i,j}$ is assumed following an i.i.d. Gaussian distribution. To enable the motion artifacts removal, basis expansion was employed to model the HR time series:

(1) $$\begin{array}{cc} y_{i,j} & =x\left(t_{i,j}\right)+\varepsilon_{i,j},\;\varepsilon_{i,j}\overset{i.i.d}{∼}N\left(0,\sigma^{2}\right), \end{array}$$

(2) $$\begin{array}{cc} x(t) & =\Phi {\left(t\right)}^{T}C, \end{array}$$

where

(3) $$\begin{array}{cc} \Phi (t) & ={\left[\phi_{1}\left(t\right),\phi_{2}\left(t\right),\cdots ,\phi_{p}\left(t\right),\cdots ,\phi_{P}\left(t\right)\right]}^{T}, \end{array}$$

(4) $$\begin{array}{cc} C & ={\left[C_{1},C_{2},\cdots ,C_{p},\cdots ,C_{P}\right]}^{T}, \end{array}$$

are the basis functions and their corresponding basis coefficients, respectively, and *P* is the total number of basis functions and coefficients. For instance, as illustrated in Figure 3, the raw HR measurements can be approximated by a functional HR curve that was formed by 13 basis functions, leading to remarkable dimension reduction and noise removal. Furthermore, the estimated functional curve is a concise representation of the HR pattern that demonstrates the trend of HR clearly, which enables personalized HR estimation. Φ(t) are pre-defined functions, such as B-spline functions and wavelet functions, and their choices can affect the performance of motion artifact removal. However, the selection of basis functions was not the focus of this paper, which aimed to provide a flexible method applicable to all different basis functions. In the case of health monitoring, B-spline functions are used to model the HR data because they are widely used in healthcare applications [25], and their differentiability conforms to the nature of HR dynamics.

### 2.3. HR Calibration by Basis-Expansion-Based Gaussian Process

To calibrate the HR patterns estimated from historical HR data with newly collected HR measurements, GP was adopted to model HR data and fuse the measurements. To enable the calibration, a calibration prior, i.e., HR baseline, needed to be constructed by utilizing the GP. Furthermore, as GP is capable of performing inference over the HR time series instead of individual HR measurements, the missed HR between any two timestamps could be estimated. The HR time series x(t) was assumed to follow a GP and could be represented as:

(5) $$\begin{array}{cc} x(t) & ∼GP(\mu (t),k(t,\tau )), \end{array}$$

where μ(t) is the mean function of GP, i.e., E[x(t)]=μ(t); k(t,τ) is the covariance function of the GP, i.e., Var[x(t),x(τ)]=k(t,τ).

Most GP models [21,27] construct the covariance function k(t,τ) by parametric models such as the popular squared exponential model: $k\left(t,\tau \right)=exp(-\frac{1}{2}|t-\tau {|}^{2}/\beta^{2})$, and assume that the mean function μ(t) is 0 by centralizing the data. However, in a health monitoring scenario, the HR time series exhibits a sophisticated correlation pattern, which is difficult to model by traditional parametric models. Therefore, we did not restrict the covariance function by certain traditional parametric models. Instead, basis expansion techniques were introduced to represent the mean and covariance functions:

(6) $$\begin{array}{cc} \mu (t) & =\Phi {\left(t\right)}^{T}C, \end{array}$$

(7) $$\begin{array}{cc} k(t,\tau ) & =\Phi {\left(t\right)}^{T}\Sigma_{C}\Phi \left(\tau \right). \end{array}$$

By combining GP and basis expansion from (2) and (5), the mean function μ(t) could be modeled as (6) for an unbiased estimation of HR time series, and the covariance function k(t,τ) could be modeled as (7) for modeling the correlation embedded in HR time series, where $\Sigma_{C}$ is the covariance matrix of basis coefficients C. To simplify the notations, several matrix-formed notations were introduced first: $T_{i}={\left[t_{i,1},t_{i,2},...,t_{i,j},...,t_{i,N_{i}}\right]}^{T}$ denotes the sampling timestamps vector, where the interval between two timestamps is unnecessary to be the same; $Y_{i}={\left[y_{i,1},y_{i,2},...,y_{i,j},...,y_{i,N_{i}}\right]}^{T}$ denotes the HR measurements vector; and $\Phi (T_{i})$ is the basis kernel matrix at timestamps $T_{i}$:

(8) $$\Phi \left(T_{i}\right)=\left[\begin{array}{ccccccc} \phi_{1}\left(t_{i,1}\right) & \; & \phi_{1}\left(t_{i,2}\right) & \; & \cdots & \; & \phi_{1}\left(t_{i,N_{i}}\right)\\ \phi_{2}\left(t_{i,1}\right) & \; & \phi_{2}\left(t_{i,2}\right) & \; & \cdots & \; & \phi_{2}\left(t_{i,N_{i}}\right)\\ \vdots \; & \; & \vdots \; & \; & \ddots & \; & \vdots \\ \phi_{P}\left(t_{i,1}\right) & \; & \phi_{P}\left(t_{i,2}\right) & \; & \cdots & \; & \phi_{P}\left(t_{i,N_{i}}\right) \end{array}\right].$$

In this BEGP model, C and $\Sigma_{C}$ are the unknown parameters that need to be estimated. Therefore, to compute the estimation of the HR mean and covariance function in (6)–(7), C and $\Sigma_{C}$ must be estimated first. Based on (1)–(7), the parameter estimators $\hat{C}$ and ${\hat{\Sigma }}_{C}$ could be derived as follows:

(9) $$\begin{array}{cc} \hat{C_{i}} & ={\left[\Phi \left(T_{i}\right)\Phi {\left(T_{i}\right)}^{T}\right]}^{-1}\Phi \left(T_{i}\right)Y_{i}∼N\left(C,\Sigma_{C}+{\left(\Phi \left(T_{i}\right)\Phi {\left(T_{i}\right)}^{T}\right)}^{-1}\sigma^{2}\right),\;i=1,\cdots ,M; \end{array}$$

(10) $$\begin{array}{cc} {\hat{\Sigma }}_{C} & =S_{C}-\frac{\hat{\sigma^{2}}}{M}\sum_{i=1}^{M}{\left(\Phi \left(T_{i}\right)\Phi {\left(T_{i}\right)}^{T}\right)}^{-1},\;E{\left(\hat{\Sigma }\right)}_{C}=\Sigma_{C}; \end{array}$$

where

(11) $$\begin{array}{cc} S_{C} & =\frac{1}{M-1}\sum_{i=1}^{M}{\left(\hat{C_{i}}-\bar{C}\right)}^{T}\left(\hat{C_{i}}-\bar{C}\right), \end{array}$$

(12) $$\begin{array}{cc} \bar{C} & =\frac{1}{M}\sum_{i=1}^{M}\hat{C_{i}}∼N\left(C,\frac{1}{M}\Sigma_{C}+\frac{\sigma^{2}}{M^{2}}\sum_{i=1}^{M}{\left(\Phi \left(T_{i}\right)\Phi {\left(T_{i}\right)}^{T}\right)}^{-1}\right), \end{array}$$

(13) $$\begin{array}{cc} \hat{\sigma^{2}} & =\frac{1}{M}\sum_{i=1}^{M}\frac{\epsilon_{i}^{T}\epsilon_{i}}{trace(I_{N_{i}}-H_{i})},\;E\left(\hat{\sigma^{2}}\right)=\sigma^{2}, \end{array}$$

(14) $$\begin{array}{cc} \epsilon_{i} & =\left[I_{N_{i}}-\Phi {\left(T_{i}\right)}^{T}{\left[\Phi \left(T_{i}\right)\Phi {\left(T_{i}\right)}^{T}\right]}^{-1}\Phi \left(T_{i}\right)\right]Y_{i}=\left(I_{N_{i}}-H_{i}\right)Y_{i}∼N\left(0,\left(I_{N_{i}}-H_{i}\right)\sigma^{2}\right), \end{array}$$

(15) $$\begin{array}{cc} H_{i} & =\Phi {\left(T_{i}\right)}^{T}{\left[\Phi \left(T_{i}\right)\Phi {\left(T_{i}\right)}^{T}\right]}^{-1}\Phi \left(T_{i}\right), \end{array}$$

are the statistics used to compute the parameter estimators. E(.) denotes expectation; $I_{N_{i}}$ is an $N_{i}\times N_{i}$ identity matrix. Among the estimators (9)–(15), $\hat{C_{i}}$ is an unbiased estimator of coefficients C for HR on the *i*th day; $\epsilon_{i}$ is a residual vector; and $\bar{C}$ is the mean of coefficients estimator $\hat{C_{i}}$ in *M* days; the sample covariance of the coefficient estimator $\hat{C_{i}}$ is denoted as $S_{C}$, as shown in (11); ${\hat{\sigma }}^{2}$ and ${\hat{\Sigma }}_{C}$ are the unbiased estimators of measurement error $\sigma^{2}$, and coefficients’ covariance matrix $\Sigma_{C}$, respectively. Consequently, the HR pattern could be estimated by the mean function in (6), and its uncertainty could be quantified by the estimated covariance function in (7), which could be employed as an HR baseline.

**Figure 4:**
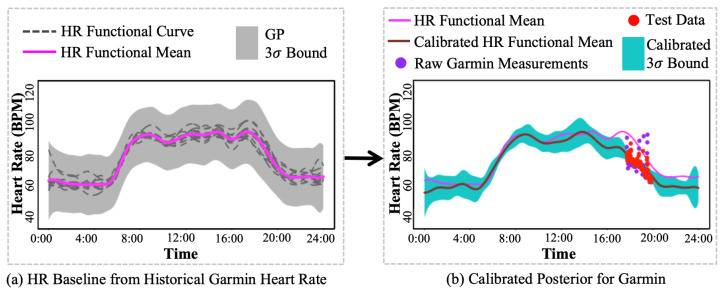
Illustration of the proposed heart rate (HR) calibration by basis-expansion-based GP (BEGP). At first, BEGP constructs the (**a**) HR baseline by estimating the HR functional mean and 3σ bound. Then, the HR base is employed to calibrate the raw Garmin HR measurements, resulting in the (**b**) calibrated HR functional mean, and narrower 3σ bound.

Constructing such an HR baseline requires a large amount of data. Thus, historical Garmin HR measurements were used to construct this prior, as shown in Figure 4a, where its mean was the functional mean learned from historical Garmin HR measurements, and 3σ bound provided uncertainty quantification of possible HR range. Furthermore, such an HR baseline could be corrected by newly collected HR measurements to improve the accuracy of HR patterns, which was proposed in the next section.

### 2.4. HR Pattern Calibration by Gaussian Process Posterior Updating

Although the HR baseline could be estimated by the proposed BEGP, it could be further calibrated for more accurate estimation on a daily basis if newly collected HR measurements were available. The calibration process could be performed under the Bayesian inference framework, where the HR baseline, including an estimated HR pattern in (6) and its covariance in (7), could be adopted as the prior of GP model:

(16) $$\begin{array}{c} x\left(t\right)∼GP(\Phi {\left(t\right)}^{T}C,\Phi {\left(t\right)}^{T}\Sigma_{C}\Phi \left(\tau \right)), \end{array}$$

where its coefficient parameter C was estimated in (9). With newly collected HR measurements $Y^{*}$ available, the coefficient parameter could be updated for a more accurate estimation, which is shown as follows:

(17) $$\begin{array}{cc} C_{i}|T_{i},Y_{i}^{*} & ∼N(C_{i}^{*},\Sigma_{i}^{*}) \end{array}$$

(18) $$\begin{array}{cc} C_{i}^{*} & =C+\Sigma_{C}\Phi \left(T_{i}\right){\left[\Phi {\left(T_{i}\right)}^{T}\Sigma_{C}\Phi \left(T_{i}\right)+I_{N_{i}}\sigma^{2}\right]}^{-1}\left(Y_{i}^{*}-\Phi {\left(T_{i}\right)}^{T}C\right), \end{array}$$

(19) $$\begin{array}{cc} \Sigma_{i}^{*} & =\Sigma_{C}-\Sigma_{C}\Phi \left(T_{i}\right){\left[\Phi {\left(T_{i}\right)}^{T}\Sigma_{C}\Phi \left(T_{i}\right)+I_{N_{i}}\sigma^{2}\right]}^{-1}\Phi {\left(T_{i}\right)}^{T}\Sigma_{C}, \end{array}$$

resulting in an HR posterior:

(20) $$\begin{array}{c} x^{*}\left(t\right)∼GP\left(\Phi {\left(t\right)}^{T}C^{*},\Phi {\left(t\right)}^{T}\Sigma_{C}^{*}\Phi \left(\tau \right)\right). \end{array}$$

As shown in Figure 4b, the HR functional mean from the HR baseline shifted towards the test data, i.e., Polar H10 measurements, and achieved a calibration posterior, including an updated HR functional mean for accurate HR pattern estimation and a narrower 3σ bound of possible HR for smaller uncertainty.

To improve the accuracy of the HR patterns estimation, the HR baseline estimated from historical Garmin HR measurements was considered as a calibration prior, then fused with newly collected Garmin measurements under the posterior updating process (16)–(20) of proposed BEGP. Consequently, the calibrated HR functional mean was balanced between the HR functional mean from HR baseline and the newly collected HR, and could be considered as a refined estimation of the HR pattern. To evaluate the effectiveness of the proposed calibration approach, a few test points from Polar H10 were randomly selected as HR ground truth, and the results are shown in Section 3.

## 3. Results

In this section, the results of artifact removal by basis expansion, as well as the personalized HR pattern estimation, are demonstrated in Section 3.1, and the results of Garmin measurements calibration are shown in Section 3.2.

### 3.1. Motion Artifact Removal and Personalized HR Pattern Estimation

**Figure 5:**
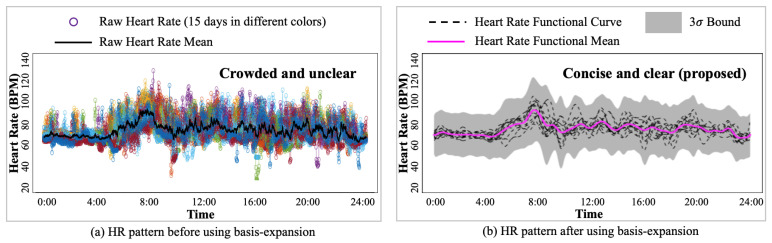
Comparisons between the (**a**) overlays of raw heart rate (HR) patterns recorded over 15 days from subject #10, with each day coded with a different color. HRs are crowded and HR patterns difficult to interpret, and (**b**) the application of the corresponding HR functional curves by basis expansion, HR functional mean, and 3σ bound by the basis-expansion-based Gaussian process, the HR trends became concise and clear to visualize.

To demonstrate the effectiveness of motion artifact removal by basis expansion, a comparison between raw HR and HR functional curves is visualized in Figure 5. Specifically, subject #10’s 15 days raw Garmin HR measurements and their mean over 15 days are shown in Figure 5a. Although raw HR mean provided a trend of HR pattern, it included excessive variations potentially induced by motion artifacts. The motion artifact embedded in raw HR overwhelms the underlying HR pattern, making the true HR pattern unrecognizable. In Figure 5b, after removing the noise induced by motion artifacts, the HR functional curves clearly describe the trend of HR pattern, and the 3σ bound quantifies the regular variation of HR.

**Figure 6:**
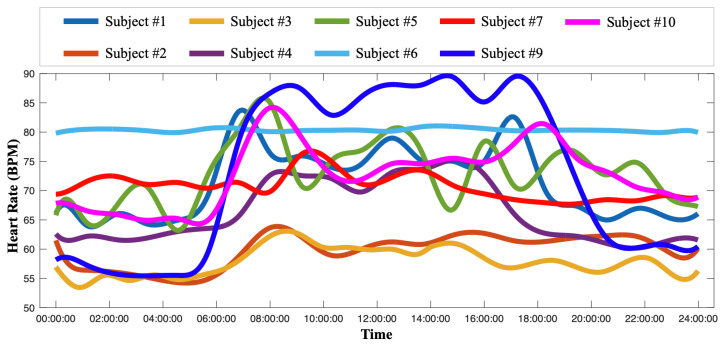
Visualization of subject-specific heart rate (HR) patterns that can be considered as personalized HR baselines.

With the basis expansion approach, subject-specific HR patterns were estimated as functional curves that could be further compared, as shown in Figure 6. By comparing the subject-specific HR patterns, the HR baseline was found to be significantly different among subjects. Thus, to achieve personalized healthcare, it is imperative to construct an individualized HR baseline for accurate monitoring, as traditional cut-off values were improper for all subjects. Consequently, these personalized HR baselines can serve as a foundation for tailoring healthcare intervention to the needs of each individual. The HR pattern for subject #6 was extremely stable as the subject was implanted with a pacemaker. Furthermore, some of the subjects shared similar HR patterns. For instance, subject #2 and subject #3 could be monitored as a group, whilst subject #1 and subject #9 could be another group. Such groups provide opportunities for tailoring group-specific healthcare and HR monitoring for subjects who share similar HR patterns.

### 3.2. HR Pattern Calibration

To evaluate the performance of the proposed HR calibration method, the HR data from the calibrated HR functional means were compared with Polar H10 HR measurements which were considered as ground truth of HR. To extend the comparison, a few benchmark methods were also compared and listed as follows:

- Method A: raw Garmin HR measurements;
- Method B: population mean of all subjects;
- Method C: subject-specific mean;
- Method D: functional mean of subject-specific HR;
- Method E: functional mean of calibrated HR. (proposed method)

**Figure 7:**
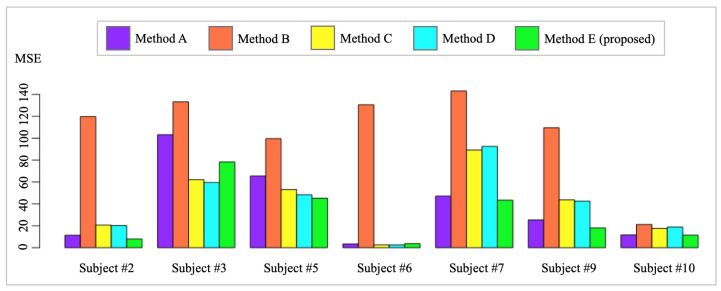
Bar plot of mean squared error (MSE) to evaluate the heart rate (HR) estimation performance of all methods for each subject. The MSE is calculated by comparing the HR estimation of each method and ground truth (Polar H10 HR), where the best method provides the smallest MSE.

In order to demonstrate the performance of HR modeling, HR values from each method were compared with Polar H10 HR measurements, which are treated as ground truth HR in this paper. Specifically, Polar H10 HR measurements from a testing day of each subject were compared with estimated HRs from all methods. The testing days are the days of Polar H10 data collection. For each subject, the historical Garmin HR data were set as the days of data collection except the testing day. For instance, the subject #2 has 16 days of Garmin HR recording, as shown in Table 1. After excluding the testing day, the remaining 15 days of Garmin HR are employed as the historical Garmin HR data of subject #2. By utilizing these subject-specific historical data, the discrepancy in HR patterns between subjects can be eliminated. However, the availability and amount of subject-specific historical data depends on the number of days of data collection for each subject, as shown in Table 2. After iterating the aforementioned process over all the testing days, the mean squared error (MSE) and error standard deviation (Error SD) for each subject could be calculated, which is visualized in Figure 7. The detailed MSE and Error SD are shown in Table 3. HR estimation was unable to be performed for three subjects (#1, #4, #8) due to the unavailability of HR ground truth obtained by the Polar H10. Thus, the HR estimation performance for the three subjects cannot be evaluated because of lacking HR ground truth, i.e., Polar H10 HR measurements.

**Figure 8:**
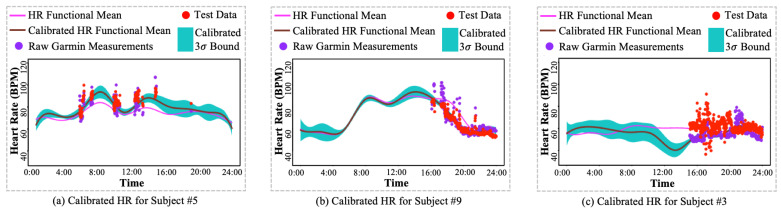
Comparison of heart rate (HR) calibration results for (**a**) subject #5, (**b**) subject #9, and (**c**) subject #3.

By comparing the HR estimation MSE across each subject, Method E (proposed) has the best performance among all subjects except subject #3 and subject #6, as shown in Table 3. Subject #6 was implanted with a pacemaker, leading to extremely stable HR measurements, as shown in Figure 6. Therefore, the subject’s HR mean is capable of providing accurate HR estimation. Method E failed to achieve the best HR estimation performance due to two factors:

- I.: Large variations from Polar H10 HR measurements. The Polar H10 HR measurements are selected as the HR measurements ground truth because of its use of ECG to allow accurate elimination of artifacts, which offers high precision and accuracy. As shown in Figure 8a,b, the Polar H10 HR measurements, i.e., test data (red dots), provide more precise and accurate HR measurements than raw Garmin HR measurements (purple dots). However, as shown in Figure 8c, subject #3’s Polar H10 HR measurements have much higher variations than the Garmin HR measurements. Such abnormal Polar H10 HR measurements can be caused by the sensor displacement or malfunction, which needs to be further investigated.
- II.: Large HR magnitude discrepancy between Polar H10 HR measurements and Garmin HR measurements. The proposed method was developed to mitigate the impact of missing values and large variations from the wearable devices. As shown in Figure 8a,b, the calibrated HR functional mean provides a complete and precise HR estimation by the proposed method. However, as shown in Figure 8c, most of the raw Garmin HR measurements are much lower than the test data, i.e., Polar H10 HR measurements, leading to an underestimation of HR by the proposed method. The large HR magnitude discrepancy between Polar H10 HR measurements and Garmin HR measurements may be caused by the sensor inaccuracy or malfunction, which cannot be solved by the proposed method.

Consequently, method E is not the optimal method for subject #3’s HR estimation, because of factors I. and II., which should be examined further.

The overall MSE provides a comprehensive evaluation of the HR estimation performance from different methods. By comparing Method A and Method E, the smaller MSE of Method E indicated the superiority of the proposed method over the raw Garmin measurements in terms of HR accuracy, as the proposed method fused raw Garmin measurements and HR baseline to obtain a more accurate HR estimation. Furthermore, the proposed calibration method provided a more accurate HR value, i.e., calibrated HR functional mean, and a possible range of HR, i.e., calibrated 3σ bound. By comparing Method B and Method C, the significantly smaller MSE of Method C indicated the necessity of personalized monitoring, where each subject is supposed to have a personalized HR baseline. By comparing Method C and Method D, Method D achieves a smaller overall MSE, which demonstrates the importance of reducing motion artifacts in the HR baseline construction, while an overly smooth functional curve may lose the details of the HR pattern, leading to a larger MSE in some subjects. In this study, Method D was used to estimate the overall personalized HR pattern instead of providing exact HR measurements. By comparing Method D and Method E, Method E has the smallest overall MSE and overall Error SD, which indicates the effectiveness of the proposed BEGP. Specifically, learned from historical Garmin measurements and newly collected measurements, the HR baseline successfully calibrated the HR pattern and achieved the most accurate and precise HR with the smallest overall MSE and smallest overall Error SD.

## 4. Discussion

In this study, we aimed to construct personalized baseline norms for continuous HR monitoring using wearable sensors with Bayesian inference techniques. The initial development of wearable sensors has significantly expanded our capacity for real-time and continuous measurements, providing an abundance of data points. Recent studies have focused on the analysis of HR variability (HRV). By analyzing HR data measured by wearable sensors and extracting signals and features from PPG or ECG, these studies have become valuable screening or diagnostic tools in various clinical specialties, such as screening for diabetes, myocardial infarction, and sleep apnea [28]. However, it is crucial to acknowledge that the reliability of HRV information relies on the integrity of the original HR data. A missing data rate exceeding 20% may lead to the risk of estimation errors exceeding 20% [29]. In the context of analyzing real-world data, which is often chaotic, heterogeneous, and prone to various measurement errors, the quality of real-world data is lower than the data collected in controlled laboratory environments. This may result in misleading or erroneous conclusions [30]. Our study addresses these challenges by employing the refined GP model, specifically the BEGP model, to estimate missing HR data. This approach quantifies uncertainty in filling data gaps and diverges from traditional single summary statistics (e.g.: mean, median, and mode), aligning more closely with the principles of precision medicine. Furthermore, despite the lower activity levels typically observed in elderly populations compared to younger counterparts, suggesting a potentially reduced likelihood of PPG artifacts due to activity, our findings reveal that artifacts persist and pose challenges for constructing personalized HR trend curves in older adults. Our study has several limitations. Firstly, the sample size of this study is relatively small. However, each participant underwent two weeks of continuous biophysiologic monitoring with HR measured simultaneously by Polar H10 and Garmin watch. Previous studies have indicated that a minimum of 8 days of observational data is required to achieve a reliability of 0.8 [31]. The amount of observation data collected in this study should, therefore, allow for reliable estimates of individual trends. Additionally, the study sample only consists of elderly individuals. While we validated the feasibility and effectiveness of this method in this specific cohort, it is important to recognize that the performance of this approach may be influenced not only by the amount and pattern of missing data but also by inter-individual physical variability. Therefore, extrapolating these results to different population groups or clinical settings requires careful validation. In future research, we plan to explore the performance of this method across diverse age groups, encompassing various health conditions and activity levels, to determine its broader utility and potential limitations. By conducting these investigations, we aim to enhance the generalizability and applicability of our findings beyond the scope of our current study. Additionally, we aim to further explore GP-estimated HR data in HRV analysis, conducting a comparison study of HRV outcomes with different methods for filling in missing HR values. Moreover, while this study constructs individual daily HR baseline norms through Bayesian inference, linking these norms to longitudinal health trajectories is an intriguing area for exploration. For instance, this could be investigating whether HR data exceeding an individual subject’s norm could serve as an early warning signal for change in health conditions, or exploring associations between changes in the norm curve and specific diseases [32]. These avenues represent important areas for in-depth research and potential clinical applications.

## 5. Conclusions

In this paper, a BEGP method is proposed to improve the data accuracy of wearable PPG sensor HR measurements. Specifically, basis expansion is utilized to estimate HR patterns as functional curves by removing excessive noise that is potentially induced by motion artifacts. The estimated functional curves can be employed to estimate the personalized HR baseline. The discrepancy of personalized HR baseline among different subjects indicates the importance of tailoring personalized health care. Among the personalized HR baseline, some of the subjects share similar HR patterns and can be grouped. These subject groups provide the opportunity to design tailored healthcare monitoring strategies for subjects who share similar biophysiological variable patterns. Additionally, once the personalized HR baseline is established, it can serve as an early warning sign for potential health events or aid in assessing the risk of developing specific diseases. For instance, if newly monitored HR exceeds the established baseline range, it could indicate the occurrence of new infection events. Further research into this methodology will not only validate the efficacy of BEGP in analyzing HR signals across diverse groups but also encompass the exploration of BEGP’s applicability to various other biophysiological signals beyond HR, including EEG, respiratory rate, and body temperature, among others.

If newly collected HR measurements are available, BEGP can serve as a calibration method to correct the HR baseline constructed from wearable PPG sensor historical HR measurements by fusing the newly collected HR measurements under a Bayesian inference framework. In this study, a wearable PPG sensor, i.e., Garmin, was employed for older adults’ HR monitoring, and an ECG sensor, i.e., Polar H10, was utilized to provide the HR ground truth. Several benchmark methods are compared for HR estimation, and the proposed calibration method achieves the smallest overall MSE and Error SD, which implies the effectiveness of calibrating HR patterns for accurate HR estimation. Furthermore, the subject-specific mean obviously outperforms the population mean, which shows the necessity of constructing a personalized HR baseline. Overall, this paper provides a new method for estimating personalized HR baseline and proposes a BEGP model for improving the data quality of HR measurements from the wearable PPG sensor. 

## Figures and Tables

**Table 1:** Subjects’ demographics and their health conditions.

Characteristic	Value (10 Subjects)
Age	79.6 (5.7) * years old
Sex	5 male, 5 female
Ethnicity	Number of Subjects (Percentage)
Hispanic or Latino	3 (30%)
White	3 (30%)
Black or African American	2 (20%)
American Indian or Alaska Native	1 (10%)
Asian American	1 (10%)
Health Condition	Number of Subjects (Percentage)
High Blood Pressure	8 (80%)
Hypotension	2 (20%)
Dyslipidemia	7 (70%)
Ischemic/Coronary Heart Disease	2 (22%); one missed
Diabetes	4 (40%)
Chronic Kidney Disease	6 (60%)
Hypothyroidism	4 (40%)
Heart Failure	1 (11%); one missed
Depression	5 (50%)
Dementia/Alzheimer’s Disease	7 (70%)
Chronic Obstructive Pulmonary Disease	1 (10%)
With Medicine of Changing Heart Rate	5 (50%)

*: Mean (standard deviation).

**Table 2:** Missing data rates and days of data collection for all subjects with ID (#number) from the Garmin device.

Subject ID										
	**#1**	**#2**	**#3**	**#4**	**#5**	**#6**	**#7**	**#8**	**#9**	**#10**
Garmin Missing Data Rate	0.4209	0.1700	0.1809	0.2709	0.1150	0.3076	0.2371	-	0.1725	0.2617
Days of Data Collection	16	16	15	12	15	14	15	-	13	16

- Dash means no record for the subject.

**Table 3:** Comparison of heart rate (HR) estimation performance of all methods for each subject based on mean squared error (MSE) and error standard deviation (Error SD).

Subject ID	Method A	Method B	Method C	Method D	Method E
#2	11.36 (1.96) ^§^	119.78 (3.26)	20.66 (2.35)	20.25 (2.28)	7.92 * (1.63) *
#3	103.19 (6.80)	133.18 **(5.39) **	62.10 (5.75)	**59.56** (5.67)	78.31 (6.16)
#5	65.48 (4.50)	99.63 (5.21)	53.10 (3.96)	48.40 **(3.82)**	**45.12** (4.27)
#6	3.42 (1.66)	130.60 (3.74)	2.57 **(1.45)**	**2.50** (1.46)	3.74 (1.57)
#7	47.07 **(4.62)**	143.14 (5.76)	89.17 (5.40)	92.53 (5.22)	**43.42** (4.64)
#9	25.41 (3.65)	109.60 (3.62)	43.67 (3.98)	42.55 (3.91)	**17.98 (2.74)**
#10	11.59 (2.66)	21.20 (3.52)	17.58 (2.81)	18.75 (2.79)	**11.46 (2.41)**
Overall	49.15 (5.31)	97.32 (5.53)	42.48 (4.61)	40.13 (4.38)	**35.83 (4.25)**

*§* Values in parenthesis are error SD. * Bold values indicate the best MSE or best error SD.

## Data Availability

The data presented in this study are available upon request from the corresponding author due to subjects’ privacy.
